# Non-spouse companions accompanying older adults to medical visits: a qualitative analysis

**DOI:** 10.1186/s12877-019-1098-y

**Published:** 2019-03-15

**Authors:** Orla C. Sheehan, Anita L. Graham-Phillips, John D. Wilson, Deidra C. Crews, Cheryl L. Holt, Jennifer Gabbard, Katherine C. Smith, Jennifer L. Wolff, David L. Roth

**Affiliations:** 1 Johns Hopkins University School of Medicine, Division of Geriatrics and Gerontology, Baltimore, MD USA; 2 THREAD Community Research Institute, Baltimore, MD USA; 3 Johns Hopkins University School of Medicine, Division of Nephrology, Baltimore, MD USA; 4 Department of Behavioral and Community Health, School of Public Health, University of Maryland, College Park, MD USA; 5 Wake Forest University, Section of Gerontology and Geriatrics, Winston-Salem, North Carolina USA; 6 Department of Health, Behavior, and Society, Johns Hopkins Bloomberg School of Public Health, Baltimore, MD USA; 7 Department of Health Policy and Management, Johns Hopkins Bloomberg School of Public Health, Baltimore, MD USA; 8 Center on Aging and Health, Johns Hopkins University, 2024 E. Monument Street, Suite 2-700, Baltimore, MD 21205 USA

**Keywords:** Medical visit companion, Qualitative research, Older persons, Family caregiver

## Abstract

**Background:**

Medical Visit Companions (MVCs) are encouraged for older adults’ routine medical encounters. Little data exist on the experiences and contributions of non-spouse companions for the growing population of older adults without a living spouse.

**Methods:**

We conducted six focus groups with forty non-spouse MVCs identified through churches in Baltimore, Maryland. Thematic analysis was used to identify key issues before the visit, during the visit itself, after the visit, and in the overall companion experience.

**Results:**

MVCs described their experiences positively but also highlighted many challenges related to the role that extended far beyond the visit itself. These included scheduling, transportation, communication, and coordination of care expectations.

**Conclusion:**

Our increasingly complex healthcare system can be challenging for older adults to navigate successfully. The diverse nature of tasks performed by companions in this study highlight the many benefits of having a companion accompany older patients to medical visits. The positive experience of the companions studied and their willingness to continue their role in the future highlights the untapped potential for increased social facilitation to improve the quality of healthcare visits and achieve patient-centered care for all older patients.

## Background

The United States National Institute on Aging (NIA) and the Agency for Healthcare Research and Quality (AHRQ) quality improvement initiatives include the recommendation that patients should bring a companion to medical visits [1, 2]. The “Medical Visit Companion” (MVC) is usually a spouse [3], however, only half of the older population now live with a spouse and this proportion continues to fall with age [4]. In the absence of a spouse, the MVC role is often filled by adult children, other family members, closer friends or community volunteers, if it is filled at all. MVCs often take on multiple roles in the visit [5, 6], acting as patient advocates, assisting with information exchange and dissemination, medication management, helping patients retain important information, and the coordination of care when needed [3, 6].

The presence of a companion favorably influences physician and patient understanding [6–9] and physicians view their presence as a positive influence in a consultation [10]. Although most companion behaviors are helpful, companion involvement can also raise challenges [7]. MVCs may have a negative impact if they serve to decrease patient responsiveness to questioning or prevent topics from being raised by the patient [11, 12]. Consultations that include family caregivers may require more time and energy on the part of provider [13] or the patient-physician interaction time may be reduced if a caregiver is also interacting with a physician [14].

As healthcare for older adults continues to become more complex, vulnerable older people often struggle to navigate care across multiple providers [15], resulting in many MVCs assuming a care coordination role. Even though policy guidelines continue to recognize the need for high quality care coordination [16], data are limited on how to best support and include MVC in care coordination roles. There is a growing appreciation of families’ relevance to patient care, however, specific knowledge is limited regarding what attributes of their involvement are valued or helpful to patients or are efficacious for improving outcomes. New initiatives to address patient concerns about possible deficits in care quality [17] seldom consider the experiences and capabilities of MVCs during face-to-face medical encounters even though such experiences may represent an important quality of care indicator [18–20]. Most existing studies of MVCs include mainly white patients and focus on spouse MVCs [6, 7, 21, 22]. There are few studies of African American companions [23] or that are targeted to understanding the experiences of non-spouse MVCs.

Social facilitation, the tendency for people to perform differently in the presence of others may also extend to experience. The presence of a companion at a healthcare visit has the potential to improve healthcare efficiency and aid in the goal of achieving patient centered care. A better understanding of the role of MVCs and increasing their use could improve the quality of healthcare received by older adults. In a qualitative study conducted in partnership with a local community-based organization, we aimed to learn more about the roles and experiences of non-spouse MVCs before, during and after a healthcare visit.

## Methods

### Participant selection and setting

Focus groups were conducted in churches in the Baltimore, Maryland. We employed a Community Based Participatory Research (CBPR) approach [24] collaborating with The THREAD (THeory, Research, Education And professional Development) Institute, a non-profit, faith-based organization focusing on mental and physical health disparities. Through this approach, we aimed to increase our shared knowledge and understanding of the MVC experience with the ultimate goal of integrating the knowledge gained with interventions and social change to improve the health and quality of life of community members. The director of the THREAD institute (AGP) was involved at all stages of the project from conception to editing of the manuscript. She helped us to reach people within the community but also to identify relevant questions and issues as well as interpret responses. We helped THREAD to achieve their goal of conducting research by providing access to our research team, taking the lead on drafting ethics approval, protocols, consent forms and interview guides, and giving them the opportunity to disseminate information through manuscripts and community and academic presentations. THREAD recruited the churches, all of whom identified with a Baptist denomination and had at least 50 adult members. We felt that basing our work in a healthcare or academic setting would not allow us to understand many of the social and economic complexities that often motivate behavior. Baptist churches in Baltimore have congregations predominantly made up of either Caucasians or African Americans allowing us the opportunity to look for differences in experience by race and provide participants with a familiar environment in which to share their experiences. Church leaders from ten churches were contacted and invited to participate. Seven churches agreed to participate by hosting a focus group. One focus group was cancelled on multiple occasions due to inclement weather, yielding three predominantly African American churches and three predominantly white churches that participated. An honorarium of $20 was provided to each participant by the THREAD Institute.

### Focus groups

Using mainly church announcements and personal contacts, adults who had accompanied an older person who was not their spouse to a healthcare visit were invited to participate in a group discussion about their experiences. Focus groups comprised of five or more participants were conducted in each church between November 2015 and February 2016 led by two facilitators, one from the THREAD Institute and one from the Johns Hopkins School of Medicine. Participants were not known to the moderators prior to the focus groups. All members of the research team contributed to the interview guide. The lead moderator (JDW) had over twenty years of experience moderating focus groups. The second moderator took field notes. Each focus group discussion lasted between sixty and ninety minutes. When moderators felt that no further themes were emerging during the focus groups data collection ended.

All participants signed an informed consent form prior to participation and the study was approved by the Johns Hopkins Institutional Review Board (IRB00037650). The relationship of the companion to the person they accompanied was noted and a unique identifier was assigned to each participant voice prior to the commencement of the focus group. The focus groups were audio recorded and later transcribed verbatim. Tapes were destroyed after transcription. Transcripts were not shared with focus group participants. The focus group interview guide is shown in Table 1.

**Table 1:** Focus Group Interview Guide

Before the visit – preparation.	
o How do you prepare for the visit? Prompts: Do you discuss the visit with the older person beforehand? Do you identify what issues do they want to discuss with the doctor? Do you check if caregivers noticed any new issues/changes? Do you write down questions? o If you do prepare do you find it helpful?	
The visit itself – procedures, usefulness.	
o Can you tell us a bit about your role during the visit itself? Prompts: - Do you take notes? - Do you include the older person in the discussion or do you/they feel that it’s more helpful for you to do the talking? - Do you normally understand everything the doctor says? - Do you feel your presence is helpful? - What do you bring with you? List of meds? List of questions? - How many people usually accompany your relative/friend? o What do you think are the important things to discuss? Prompts: medical issues? medications? function? o Can you suggest ways in which the setup of the consultation could be improved? o How often do you accompany someone to visits – is it always the same person?	
After the visit – the overall experience, welcome, willingness to do it again, disseminating information learned at visit.	
o What benefits, if any, do you get from accompanying someone to a medical consultation? o What impact, if any, does accompanying someone to a medical consultation have on you? o Do you feel welcome? o Are any groups of physicians more welcoming? Gender, specialty. o Do you disseminate the information to other family members, caregivers etc.? o Why do you accompany people? o Would you like more information on accompanying someone to a medical consultation? o Do you plan on being a Medical visit Companion again in the future?	

### Qualitative data analysis

To examine the data from the focus group transcripts, thematic content analysis was used [25]. One project team member (AGP) who had extensive experience as well as formal training in qualitative methodology and coding independently reviewed all transcripts for initial themes and patterns [26]. All team members involved in coding were sent the transcripts and a list of the initial themes identified by the lead coder for review prior to meeting. The four coders then met over the course of a day reviewing transcripts both alone and in groups to build upon the list of themes. Many themes fell into three major categories of roles performed by companions based on timing before, during and after the visit e.g. transport emerged as a theme in the discussion around roles before the visit. Any remaining themes were captured by the concept of companion experience. A preliminary consensus list of these four major themes was created and within each theme many separate codes emerged [27, 28]. A codebook was then developed defining each theme and identifying representative passages from the data [29]. All coders independently coded two transcripts to trial the codebook then reviewed their results with the group and codes were refined and aggregated as needed. Once consensus was reached on the codebook, coding of all transcripts began. All transcripts were reviewed by two coders (AGP and OS, JG and JW). Each coding pair coded all transcripts for two of the organizing themes. As part of our CBPR approach each coding pair was comprised of an academic partner representative and a community partner representative. One coder in each pair was Caucasian and the other was African American. This allowed coders the opportunity to bring their own cultural perspectives to the analysis, to propose codes that perhaps would not have been identified by a coder of a different racial group to the focus group participants and improved our ability to interpret nuances of language, phraseology and spirituality. Discrepancies were resolved through discussion in a process of constant comparison. Inter-rater agreement rates [30] ranged from 85.3 to 94.1%. Representative quotes to illustrate each code were collected. Thematic saturation was largely achieved after four focus groups with only one new code emerging in the fifth group and none in the sixth. The participant identifiers allowed us to consider cultural sensitivities within the data when we conducted the analyses.

### Participant demographic data

Focus group participants completed a brief demographic questionnaire that included questions on gender, race, age, education, income, employment, the caregiver’s relationship to the patient, the medical condition of the patient and if the participant provided any help to the patient with Activities of Daily Living (ADLs) or Instrumental Activities of Daily Living (IADLs). Questionnaire data were compiled to describe the participant sample.

## Results

### Demographics

Forty non-spouse MVCs participated in the focus groups of which 24 participants (60%) were African-American (AA), 1 (2.5%) was Asian and 15 (37.5%) were White. The mean age of MVCs was 50.1 years (SD 11.5) and of the patients they accompanied was 77.7 years (SD 20.1). Many of the MVCs (52.5%) reported accompanying more than one older adult to healthcare visits, with 5 participants accompanying four or more people within the previous year. Most companions were the adult children (*n* = 32) of the person or were related in another way including siblings (*n* = 9), grandchildren (*n* = 4) and other relatives (*n* = 7). Four companions were friends and three did not have a family relationship with the person they accompanied but responded to a call from their church to accompany a person who otherwise would be alone. Descriptive characteristics are shown in Table 2. White participants were more likely to be employed than AA participants (61.1% vs. 16.1%, *p* = 0.0004) but no race differences were seen by age, income, relationship status or education.

**Table 2:** Demographics of focus group participants

Variable	N (%)
Female	37 (92.5)
Age mean	50.1
Hispanic n (%)	1 (0.03)
Income under $50,000	9 (22.5)
Currently in a relationship	20 (50.0)
Currently employed	15 (37.5)
High school education or less	12 (30.0)
ADLs/IADLs for which assistance is provided, Number mean	5.4
Provides assistance with dressing	20 (50.0)
Provides assistance with bathing	20 (50.0)
Provides assistance with toileting	15 (37.5)
Provides assistance with mobility	19 (47.5)
Provides assistance with feeding	15 (37.5)
Provides assistance with bed transfers	14 (35.0)
Provides assistance with grooming	17 (42.5)
Provides assistance with meal preparation	25 (62.5)
Provides assistance with housework	21 (52.5)
Provides assistance with finances	12 (30.0)

*N* number

Accompanied persons had a wide range of medical conditions and disabilities including chronic pain (30.6%), visual or hearing loss (26.5%), dementia (26.5%), stroke (18.4%), heart disease (18.4%), diabetes (22.5%), cancer (16.3%) and kidney disease (4.1%). In addition to accompanying the patients to healthcare visits, 90.0% of participants also provided informal caregiving duties including help with activities of daily living (ADLs) and instrumental activities of daily living (IADLs) as described in Table 2.

### Role of the MVC before the visit

#### Transport

Participants spent time on logistical planning before the appointment and transportation was identified as an important issue for many MVCs. Organizing transport, fitting equipment in cars, getting the patient in and out of the car, and parking in a convenient place were all time-consuming tasks that required a lot of forward planning. One participant described how it could take an hour just to get into the car as she had to load her mother, her mother’s scooter and her own wheelchair into her van. Another participant commented:

“You have to consider the time it’s going to take… getting ready and getting out of the house. It takes time to get into the car. It takes time to get out of the car. Then it takes time to get into the place”. (ID 0504, adult child).

Participants described accompanying older adults to many different hospital-based specialists and less frequent visits to community based primary care providers. Several people expressed frustration trying to book hospital transportation and described begging administrators for help when the patient’s ability to mobilize fluctuated. One participant commented:

“Sometimes my mother could walk and sometimes she couldn’t walk” but “I’m at home trying to get her from the chair to the wheelchair and to the car, you know, and I need assistance…”. (ID 0604, adult child).

#### Gathering relevant information

Before the visit, companions described spending time gathering information, ensuring that the medication list was up to date, locating insurance cards (without which the patient would not be seen by the healthcare professional) and making lists of questions or thoughts to ask the provider. Around 60% of companions reported bringing written notes to the visit.

“Before the visit I had to make sure she had all of her cards and records. I mean there was so much to prepare”. (ID 0206, niece).

“I question (her) before the appointment just so I’m clear on what she’s experiencing and then I make a list of questions”. (ID 0303, parent of a disabled child).

#### Encouraging attendance

In each focus group companions described experiencing resistance from patients not wanting to attend an appointment. One participant commented:

“We cannot tell her she’s going somewhere until it’s time to get ready to go because if I tell her on Monday that Friday she has to go, she will build up little ailments and then by Friday and it is time to get ready to go she’s sick”. (ID 0702, adult child).

Another participant described how difficult and time consuming it was to convince her mother to attend and how tiring it was to:

“Stop all that fighting that they be doing cos they don’t want to go”. (ID 0101, adult child).

### Role of the MVC during the visit

#### Exchanging information

The MVCs saw giving and receiving information as their most important role during the visit. This information allowed them to better care and support their family member or friend. One participant described the benefit of receiving information:

“It helped me to understand better when he was having …health problems – what they were and how to deal with him when I got him back home and trying to help him follow instructions, you know, and do whatever the doctor asked him to do”. (ID 0601, adult child).

Several companions reported providing collateral information to health care professionals:

“She’s a private person and didn’t always want to open up. I had to have some history in order for me to help her answer or help her get the information to the doctor so he could help her” (ID 0204, adult child).

“Saying no Mom you really didn’t do that or Doc she’s not being honest”. (ID 0503, adult child).

#### Advocacy

Companions presented their role as patient advocates who work to promote the best interest of the patients, helping the patient understand what the doctor was saying, seeking clarification to help understand the condition and treatment and challenging providers when they felt it was necessary. Five participants reported previously witnessing what they perceived as discrimination of older persons and said that this was the main reason they felt that older people should be accompanied. One commented:

“I sort of feel that old people are discriminated against. They can wait or whatever. It’s not a big deal or whatever it is….And especially old people with dementia …If you don’t stand up and start jumping up and down you might not ever get seen” (ID 0405, volunteer).

#### Communication

Most participants reported having difficulties understanding medical language and seeking clarification. The majority pointed out how they try to do this is a non-confrontational way with one reporting:

“One of the main things that I’ll say over and over again is I didn’t go to medical school. Can you dummy (sic) that down to me?” (ID 0803, adult child).

A small number of participants reported meeting resistance when they asked questions. One participant described:

“When you asked questions she kind of looked … she was hesitant as to why she would have to explain them to you”. (ID 0303, parent of disabled adult child).

Visit summaries prepared by healthcare providers were seen as particularly helpful especially for communicating with other family members or caregivers. Difficulties with scheduling and administration rather than issues with providers were reported more frequently as reasons to leave a provider. One participant recounted how she told her mother’s physician:

“I get an attitude from the same person over and over again, I’m just not going to … you may be the best doctor in the world but I’m not going to put myself through all that”. (ID 0703, adult child).

#### Visit duration

Frustration at the short consultation time was clear but many companions felt that the system and not the provider was at fault. One participant commented:

“Once you’re on Medicare there’s certain things that he no longer can do because of this Act has passed so that has put pressure on doctors because they’re only allowed a certain amount of time” (ID 0405, adult child).

Another participant described how time pressure forced her to be assertive in order to get all of her family member’s issues dealt with during the consultation and reported saying to the provider on more than one occasion:

“I’m sorry you’re in a hurry but I’m not done. I really need to know this”. (ID 0204, adult child).

Another participant described:

“In that 5 minutes you got to try and get all the information that you got to take out of there”. (ID 0304, adult child).

### Role of the MVC after the visit

#### Dissemination of information

All companions reported carrying out visit-related tasks in the days and weeks following the visit. Participants reported spending time explaining to the patient what the providers had said and the outcome of the visit. One participant reported:

“I make sure I understand what’s going on and then in turn, make sure she understands” (ID 0108, adult child).

“I can say the doctor told us such and such and the patient very often be like “really”? You have a clearer head and can stay on target and tell them later” (ID 0504, adult child).

Information learned at a visit was commonly shared with family members. One participant when referring to the written summary of the visit often given to patients after a healthcare encounter described:

“I make copies and send it to my sisters so we’re all on the same page”. (ID 0505, adult child).

#### Support

Many companions continued to provide support, relief and emotional assistance to the patient who often became upset after the visit. It was common to hear how the accompanied person remained stoic during the visit but became upset on the way home or in the days following the visit. In most cases the distress was not caused by what the companion saw as “bad news” but by changes to an older person’s routine such as the prescription of new medications, dietary changes or the scheduling of additional tests or visits. Some companions who were also caregivers for their relative described having to deal with challenging behaviors from their loved ones after the visit. One woman described how the change in routine upset her father who had Alzheimer’s and how hard it was for her sister and her to care for him in the hours after a visit. Many participants reported spending time in the days and weeks following a visit trying to ensure that the patient followed the instructions given by the provider, with one participant commenting:

“Sometimes you have to be a detective…the patient will come home and do just what the doctor said not to do. My father had fluid retention. What he do? He come home trying to have him a ginger ale and he hides it because he knows he’s not supposed to have it…But his wife, the detective, snatch it away” (ID 0504, adult child).

#### Follow up

After the visit almost all companions reported spending time organizing follow up appointments, scheduling tests and seeking medical records from other institutions. Several participants described feeling frustrated by the lack of care coordination and the perception that different specialists weren’t communicating with one another. Difficulties scheduling follow up appointments and filling prescriptions were also common sources of frustration.

#### Perceived misdiagnosis

A small number of companions described feeling frustrated by what they perceived as misdiagnosis or delayed diagnosis which had come to light during the visit. In most cases neither the patient nor companion had discussed this with the provider or sought clarification around the issues they described. One participant described:

“I was really mad but I didn’t say anything ….I was too intimidated to ask a lot of questions ……I was so upset with the doctor but I was intimidated because I felt well he’s a lot smarter than me”. (ID 0303, parent of disabled adult child).

### Companion experience

#### Overall experience

Accompanying a patient to a healthcare provider visit was generally seen as a very rewarding experience by companions. One participant who had accompanied several people as both a paid companion and as an unpaid family member described:

“I think we get more out of it. I did…Like you said just feeling the trust, you know, knowing that I’m going to be okay. That I have someone who can help me. So that makes me feel really loved. So I feel wonderful. I feel good.” (ID 1201, niece).

Companions described feeling reassured that they knew and understood what their relative’s issues were and often reported being ready to step into a caregiving role should it become necessary:

“It’s good to be certain about the treatment and be ready for what comes” (ID 0304, adult child).

“Helped me to understand better when he was having health problems”. (ID 0301, adult child).

Several companions described learning new skills through their role with one participant remarking:

“I learned more patience, comfort and understanding of everything”. (ID 0401, granddaughter).

#### Relationship with accompanied person

Being a MVC often strengthened a person’s relationship with the person they accompanied. One participant when describing her relationship with her mother after she had accompanied her to provider visits and supported her as she received bad news commented:

“Me and her, I got a different relationship”. (ID 0701, granddaughter).

#### Negative aspects to being a companion

Although companions described how rewarding the experience was and how willing they would be to do it again, most acknowledged that there are negative aspects to being a MVC. Many worried in advance of the visit about what would happen and had concerns about what to say or do. A few companions commented on the emotional strain of preparing for the visit and described how their emotions are often fueled by the emotions of the person they are accompanying with one participant reporting:

“What causes me anxiety is usually her stress”. (ID 0603, adult child).

MVCs reported discussing possible outcomes with other family or church members in advance of the visit in an attempt to learn how best to support the person they were accompanying.

Making up lost time at work and getting leave to accompany the patient were both significant concerns for MVCs. A number of companions described times when they were very worried about the outcome of a test and how it affected their own health. Many people described good support networks with one companion remarking of her sister:

“When we get depressed or whatever, we bounce off each other”. (ID 0902 adult child).

#### Cultural diversity

Differences emerged in how companions viewed their role with focus groups of African American participants including more frequent discussions of how grateful they were for the opportunity to be a companion and to strengthen their relationship with the person they accompany. One participant described her role as:

“You have been gifted…to take that on” (ID 0401, granddaughter).

Another described:

“When you know that … they are not alone in this journey, um, it’s very rewarding” (ID 0403, adult child).

Although many white participants also described being grateful that they were able to help their loved ones they used words like “hard” and “burden” to describe their role more frequently than African American participants.

Focus groups of predominantly white participants more often included discussions of frustration and dissatisfaction after a visit than African American focus groups. Two white participants described their feelings when reflecting on the visit:

“I was so angry” (ID 0403, adult child).

“(feeling rushed by the provider) annoys me so much” (ID 0406, adult child).

This compares to an African American participant who when asked to reflect on the visit said:

“I think for us caregivers, we get more out of it. I did. Just seeing that they were comfortable” (ID 0109, adult child).

Groups of African Americans participants more frequently identified feelings of stress and anxiety about the companion role but the same groups also included more discussions around their access to a wide support network to help them to deal with the difficulties they encountered.

“Sometimes I don’t know what to do but we have a good support system between my sister and my son and her daughter… we take turns.” (ID 0402, Adult child).

## Discussion

We examined the experiences of forty non-spouse Medical Visit Companions affiliated with six Baltimore Baptist churches by conducting focus groups and studying the themes that emerged. MVCs helped with many key processes throughout the medical visit journey. They described getting the patient to the appointment on time and with the information required, sharing information with the provider, advocating on behalf of the patient, developing an understanding of the condition and treatment plan, ensuring that issues were addressed with the allotted time, disseminating acquired knowledge to relevant parties and arranging follow up appointments and tests as required.

One important finding is that focus group participants spent much of the discussion outlining their roles before and after the healthcare visit rather than focusing on their role during the visit itself. Few studies have examined the very significant role played by MVCs before and after a healthcare visit [12] with most literature focusing purely on their contributions during the healthcare encounter [3, 7–10, 14, 21, 31]. These studies typically examine the influence of the companion on the provider, the patient, or on the provider-patient interaction and report similar roles to those described by our participants; advocacy and assistance with communication and information exchange. Although much has been written about the possible health consequences of family caregiving, the specific experiences of medical visit companions have been largely overlooked even though the companion is in many cases not the traditional family caregiver but may be an adult child, friend or even community volunteer who has taken on the role of accompanying an older person to a healthcare visit. Our work sheds light both on the companion experience and on the important and often unnoticed roles played by MVCs.

A key aspect of the companion role highlighted by many participants is care coordination. The emergence of this care coordination theme may reflect the difference between spousal and non-spousal companions of older persons, with adult children and other relatives often feeling better able to take on this role than older spouses who may have healthcare issues themselves. Care coordination becomes particularly important for older adults with multiple chronic conditions [32] who often see many different providers and healthcare facilities while juggling a variety of different treatments and medical tests. With proper support and acknowledgment of their critical roles and guidance through issues around confidentiality, MVCs can help facilitate the exchange of information between providers, highlight key events in the medical history, facilitate a smooth transition between health care entities, and facilitate adherence. Through the Affordable Care Act many new models of care delivery are emerging in the United States with care coordination playing a key role. These care coordination programs allow providers and other members of the health care system to work together for the benefit of the patient and should be encouraged to include the perspective of those companions who currently fill many aspects of this role.

Few differences emerged in the MVC experience between the focus groups in the white churches and the African American churches, and those that did related to how people felt about tasks and events, not which tasks were carried out. White participants recounted more negative provider encounters than African-Americans and were frequently left frustrated after the visit. African-Americans more commonly reported positive emotions such as feelings of gratitude and love. African American caregivers typically describe less burden and a lower sense of intrusion on their lives due to caregiving than white caregivers [33]. A similar cultural effect appears to exist in MVCs. African Americans reported some negative effects such as increased stress more commonly than white participants but they did not complain about them perhaps because they were more likely to describe having access to a support network. Traditionally, African American families tend to provide care in collectivist versus individualistic caregiving systems which may explain differences in access to support networks [34, 35]. The expectations of both groups prior to the visit were not explored leaving the possibility that perceived cultural diversity was merely a difference in expectations about roles.

Despite the many challenges reported by MVCs, most described their experience as a rewarding one and expressed a strong willingness to accompany the person again in the future. This positive experience of providing help or assistance to a loved one is mirrored in the caregiving literature where new studies are challenging the traditionally negative views of the stressful aspects of caregiving [36]. A similar effect may be seen in MVCs where despite many frustrations and challenges most MVCs viewed the experience as more positive than negative.

Although most of our focus group participants were family companions the focus groups did also include three non-family companions who responded to a call from their church to accompany a vulnerable older person without any available family. These volunteers provided transportation, care coordination and much more. As the gap widens between older Americans’ need for care and the availability of family members to provide that care [37] it is likely that more volunteer MVCs will emerge through faith based organizations, senior centers and community organizations. Further research is needed to determine the acceptability and usefulness of non-family, volunteer MVCs to patients, providers and the volunteers themselves.

Limitations of our study include the potential for bias in the self-reported data of all focus groups with data limited to the contributions of those who voiced their ideas and experiences [26]. We conducted the focus groups in the community and not in a healthcare setting to encourage participants to talk freely about all aspects of the MVC experience. The church-based setting, however, may have additional limitations on the extent to which these findings apply to the general population, and it may have discouraged some participants from reporting negative feelings about their relative that they did not wish other church members to hear.

## Conclusion

It is apparent that MVCs play a diverse and often underestimated role in the older person’s medical encounter. As more and more older people lack an available family member to accompany them, many community organizations including churches are ready and willing to help but lack the specific knowledge and understanding to train their members appropriately for the MVC role. Policy makers and healthcare systems need to promote awareness of the value of MVCs among older adults, healthcare professionals and the general population but also provide the necessary information and resources to community organizations to support, educate, and prepare MVCs for their role.

## Abbreviations

AA: African-American
ADLs: Activities of Daily Living
AHRQ: Agency for Healthcare Research and Quality
IADLs: Instrumental Activities of Daily Living
MVC: Medical Visit Companion
NIA: National Institute on Aging
SD: Standard Deviation
THREAD: THeory, Research, Education And professional Development
